# Exploring the “New Normal”: Older Adults’ Coping Strategies During COVID-19 Pandemic

**DOI:** 10.1093/geroni/igab046.2679

**Published:** 2021-12-17

**Authors:** Novia Nurain, Lesa Huber, Clara Caldeira, Kay Connelly

**Affiliations:** 1 Indiana University, Indiana University, Indiana, United States; 2 School of Public Health, Indiana Univ, Bloomington, Indiana, United States; 3 Indiana University, Bloomington, Indiana, United States

## Abstract

COVID-19 stay-at-home orders resulted in social isolation and psychosocial challenges for older adults around the world. To understand their lived experiences during the pandemic, we conducted a qualitative study using semi-structured interviews with 15 older adults living in community settings. Qualitative thematic analysis of the collected data identified themes and patterns of the “new normal” for these participants: ways of living, communication with family and friends, sense of autonomy, psychological responses, coping strategies, and perceived social support. This presentation focuses on participants’ coping strategies. Participants used common coping strategies, customized to the unique challenges of stay-at-home orders. We categorized coping strategies as problem-focused, meaning-focused, and emotion-focused. Participants’ problem-focused strategies aimed to reduce the risk of infection. Meaning-focused strategies included purposeful errands such as going to grocery stores. Emotion-focused strategies emphasized connecting with support networks (e.g., via Zoom) and efforts to maintain psychosocial and emotional well-being (e.g., seeking professional counseling). They also employed self-enhancing comparisons to increase self-concept and self-esteem. At the beginning of the pandemic, some temporarily used distraction/avoidance strategies such as eating comfort food and avoiding news about COVID-19 to maintain a positive emotional state. Our findings imply the applicability of frameworks such as life course perspective and selective optimization with compensation to highlight the successful adaptive strategies developed by older adults through experience. We argue against the ageist view of older adults as vulnerable. Rather, this study suggests that older adults can flexibly employ resilient coping strategies crafted over a lifetime of experiences in response to crises.

